# Increased mROS Generation Associates With Cardiovascular Risk in BioHEART‐CT PBMCs

**DOI:** 10.1111/cts.70469

**Published:** 2026-01-18

**Authors:** W. Eugene Lee, Albert Henry, Eleanor Ruth Spenceley, Eszter Sagi‐Zsigmond, Blake Bowen, Tung V. Nguyen, Michael P. Gray, Stuart M. Grieve, Joseph E. Powell, Gemma A. Figtree

**Affiliations:** ^1^ Faculty of Medicine and Health University of Sydney Sydney New South Wales Australia; ^2^ Kolling Institute St Leonards New South Wales Australia; ^3^ Translational Genomics Program Garvan Institute of Medical Research Sydney New South Wales Australia; ^4^ Faculty of Medicine and Health University of New South Wales Sydney New South Wales Australia; ^5^ Department of Cardiology Royal North Shore Hospital, Northern Sydney Local Health District St Leonards New South Wales Australia

**Keywords:** atherosclerosis, biomarker, coronary artery disease, dysfunction, mitochondria, PBMCs

## Abstract

Coronary artery disease (CAD) remains a leading cause of morbidity and mortality worldwide, and identifying accessible blood‐based biomarkers is therefore a clinical priority. Given the involvement of oxidative stress and immune cell dysfunction in atherosclerosis, we investigated whether mitochondrial reactive oxygen species (mROS) production in peripheral blood mononuclear cells (PBMCs) is associated with CAD. This exploratory study analyzed PBMCs from 40 BioHEART‐CT participants with or without CT‐defined CAD using MitoSOX‐based flow cytometry. In parallel, single‐cell RNA sequencing (scRNA‐seq) was conducted in the same individuals to investigate differential expression of CCBE1, a recently implicated gene in cardiovascular disease, across PBMC populations. Overall, mROS levels in PBMCs and their major cellular subtypes did not show consistent or meaningful associations with CAD status or with modifiable cardiovascular risk factors. Small, subgroup‐specific signals—such as moderate association between lymphocyte mROS and coronary artery calcium score in males, and a modest inverse association between monocyte mROS and hypertension—were exploratory and not uniform across analyses. scRNA‐seq analysis did not identify a distinct CCBE1 expression signature in PBMCs. These findings indicate that PBMC‐derived mROS is unlikely to serve as a useful cross‐sectional biomarker of CAD in stable populations.

## Study Highlights

- What is the current knowledge on the topic?
  ○ Peripheral blood mononuclear cells (PBMCs) contribute to atherosclerosis, and mitochondrial dysfunction with excessive reactive oxygen species (ROS) generation has been implicated in cardiovascular disease. However, the role of mitochondrial ROS (mROS) in PBMC subsets as a biomarker for CAD has not been clearly established.
- What question did this study address?
  ○ This study examined whether PBMC‐derived mROS levels are associated with CAD, measured by coronary artery calcium scoring, and explored whether single‐cell transcriptomics could identify a distinct gene expression signature (specifically CCBE1) related to CAD in PBMCs.
- What does this study add to our knowledge?
  ○ The study provides preliminary evidence that lymphocytic mROS production may be associated with coronary artery calcium burden in male participants, suggesting a potential link between PBMC mitochondrial redox status and CAD. Additionally, elevated monocyte mROS showed a modest and exploratory association with lower odds of hypertension. These findings indicate potential cell type–specific differences in redox responses, but the observed associations have small effect sizes and require validation. No distinct CCBE1 signature was observed in PBMCs, helping refine future directions for biomarker discovery.
- How might this change clinical pharmacology or translational science?
  ○ These findings suggest that PBMC mROS profiling could contribute to understanding CAD‐related redox phenotypes. However, the current data are insufficient to support biomarker utility. If future studies with larger and longitudinal studies confirm these preliminary associations, PBMC redox phenotyping may have the potential to complement existing approaches for CAD risk assessment and for identifying individuals who could benefit from therapies targeting oxidative stress.

## 1 Introduction

Coronary artery disease (CAD) remains the leading cause of mortality and morbidity worldwide [1]. Among patients admitted for their first presentation of ST‐segment elevation myocardial infarction (MI), up to 27% present with the absence of standard modifiable risk factors (SMuRFs) in addition to suffering 47% higher 30‐day mortality when compared to those with at least one SMuRF [2, 3]. Therefore, it is of upmost importance to construct a novel blood‐based biomarker for the early detection and treatment of CAD.

Human peripheral blood mononuclear cells (PBMCs) are key drivers of immune responses, comprising several classes of immune cells, including T cells, B cells, monocytes, and natural killer (NK) cells [4]. These cells play a crucial role in both innate and adaptive immunity, responding to infections, inflammatory signals, and cellular stress [5]. As circulating immune cells, PBMCs act as excellent sentries for the health of the human vasculature, continuously monitoring and responding to pathogenic threats, tissue damage, and metabolic disturbances. Their composition and functional state can provide valuable insights into immune system dynamics and disease pathogenesis.

Mitochondria, often referred to as the powerhouse of the cell, play a central role in cellular energy metabolism through oxidative phosphorylation. Beyond their bioenergetic function, mitochondria are also key regulators of immune responses, apoptosis, and redox signaling. One of their critical functions includes the production of ROS, which serve as signaling molecules in various physiological and pathological processes [6, 7, 8]. While ROS are necessary by‐products of cellular metabolism, acting as intermediary messengers that transduce intracellular signals involving biological processes such as cell proliferation, differentiation, and immune activation, excessive ROS production can contribute to oxidative stress [7, 9]. This oxidative stress is implicated in the progression of atherosclerosis by promoting endothelial dysfunction, lipid oxidation, and inflammatory responses within the vascular wall [7].

Previously, the mitochondrial ROS (mROS) production levels of endothelial colony forming cells (ECFCs) from patients with or without CAD have been used to stratify coronary risk and burden [10]. This is due to ECFCs retaining a phenotypic memory of their disease state [6, 7, 10, 11]. More recently, this has also been translated for use as a drug discovery tool [6]. CAD is widely regarded as a chronic inflammatory condition, and we hypothesized that long‐term vascular inflammation might be reflected in circulating immune‐cell mitochondrial activity. On this basis, we therefore examined PBMC mROS within the BioHEART‐CT cohort, anticipating that stable CAD would still exhibit detectable redox alterations. Whether such chronic inflammation produces consistent PBMC mitochondrial signatures, however, has not been established. To test that, PBMCs were isolated from patients with or without CAD and treated with MitoSOX to identify mROS production levels.

## 2 Materials and Methods

### 2.1 Participants

This study included samples from the BioHEART‐CT cohort (Australia New Zealand Clinical Trials Registry: ANZTR12618001322224, Camperdown, Australia), a longitudinal, prospective, multicenter study of participants referred for clinically indicated coronary CT angiography (CCTA) [12]. Adults with previously diagnosed or suspected CAD provided written informed consent to participate. Clinical data were collected through facilitated interviews at recruitment, encompassing demographics, anthropometrics, medical history, family history, medications, occupational and exposure history, and the indication for CCTA. CAD status was determined at the time of CACS assessment, with CACS = 0 indicating no detectable calcified plaque and higher scores indicating varying burden of subclinical disease.

The sample size was determined by the availability of processed samples, and no formal power calculation was performed. As such, the study is hypothesis‐generating, and the findings should be interpreted cautiously. For mROS quantification, 40 samples were included based on consecutive patient recruitment and the availability of processed material. Because the study was not powered specifically to detect effect sizes in mROS levels, the observed variability and wider confidence intervals were expected. These analyses are intended as preliminary, motivating future validation in larger, formally powered cohorts. This study was conducted according to the guidelines of the Declaration of Helsinki and approved by the Northern Sydney Local Health District Human Research Ethics Committee (2019/ETH08376).

### 2.2 Imaging Analysis

CCTA was performed using a 256‐slice CT scanner following standard clinical protocols [12, 13]. When necessary, heart rate was optimized with oral beta‐blockers or ivabradine before the procedure. To minimize radiation exposure, current dose‐reduction recommendations [14] were strictly followed. Coronary artery calcium score (CACS) was assessed before CCTA using the Agatston method [15], which quantifies calcified plaque in the coronary arteries. An age‐ and sex‐adjusted CACS percentile was generated for each participant. CACS is a well‐validated surrogate marker of coronary atherosclerotic burden and is strongly associated with future cardiovascular events [16, 17, 18, 19]. These were used as a surrogate marker for CAD in this study.

### 2.3 PBMC Isolation

Peripheral blood samples were collected immediately after the insertion of the venous cannula required for CCTA. Lithium heparin blood tubes were used for PBMC isolation and stored at room temperature until processing. Within 4 h of collection, PBMCs were isolated using a standard Ficoll preparation method [20] and immediately used for experiments.

### 2.4 Flow Cytometry Analysis

**FIGURE 1:**
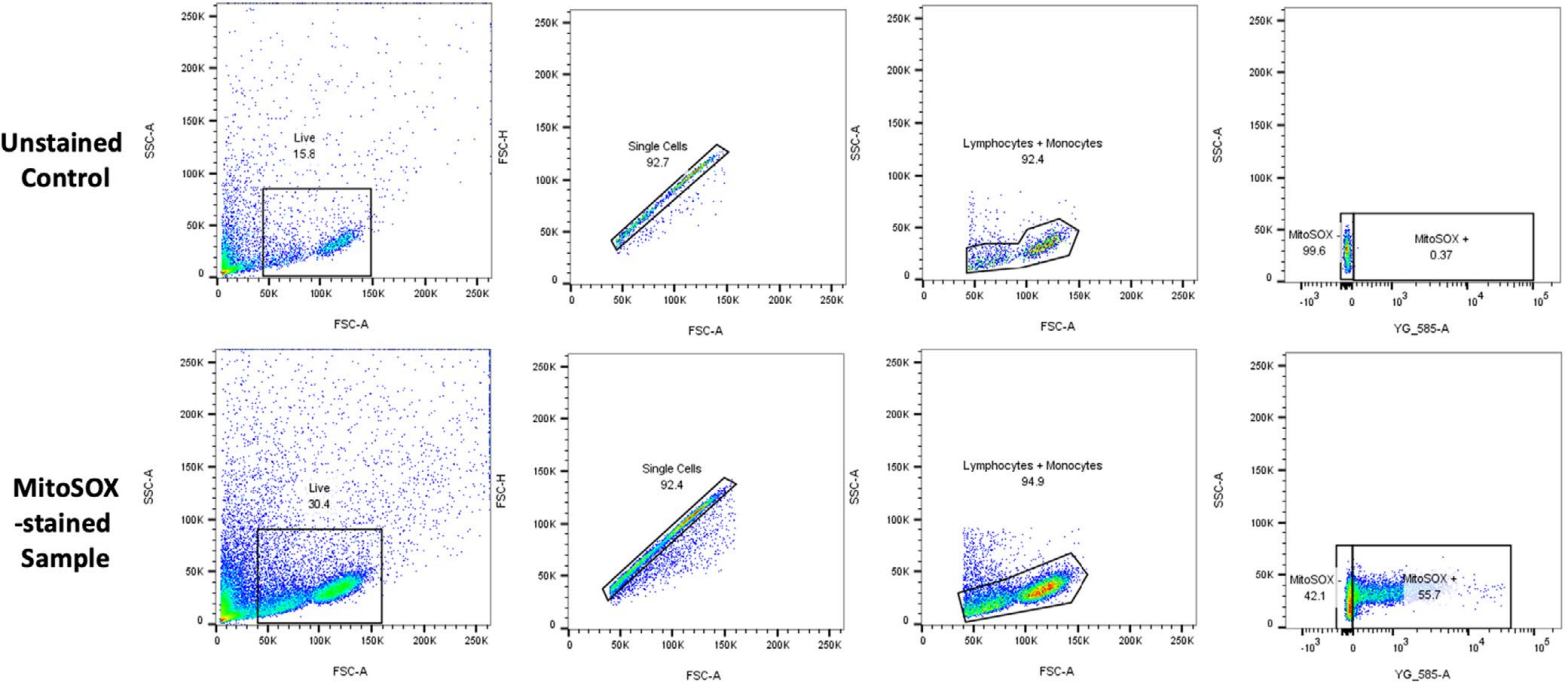
Representative gating strategy for mitochondrial ROS production (MitoSOXTM Red) in freshly isolated patient‐derived PBMCs with or without CAD.

Cells were washed with warm PBS and centrifuged at 500× g for 3 min. After removing the supernatant, cells were resuspended in fluorescent staining buffer (10% HIBS, 90% PBS) and stained with 5 μM MitoSOX Red (Invitrogen, USA) for 25 min at 37°C. They were then centrifuged again at 500× g for 3 min and resuspended in Fluorobrite DMEM (Invitrogen, USA). Until analysis, cells were kept on ice in the dark. Flow cytometry was performed using the LSRFortessa (BD Science, Australia), with MitoSOX Red excited at 561 nm and detected using 585 nm. Single‐stain and no‐stain controls were included for compensation. A minimum of 10,000 events was acquired per sample, and data were analyzed using FlowJo (FlowJo LLC, Ashland, OR, USA). Our gating strategy to quantify live PBMCs, lymphocytes, and monocytes started with discrimination of cells by size (FSC‐A by SSC‐A). Singlets were distinguished using FSC‐H by FSC‐A (Figure 1).

### 2.5 Single Cell RNA‐Seq of BioHEART‐CT Patients

Detailed methods have been described previously [21]. Briefly, we leveraged data from Phase 1 of the TenK10K project, which includes 5.4 million single cells from 1925 individuals of European ancestry [21]. This dataset integrates deeply phenotyped participants from the BioHEART‐CT study—a cohort of 1356 individuals undergoing coronary CT angiography (CTCA). Following rigorous QC, PBMCs of BioHEART‐CT participants that were used for MitoSOX flow cytometry were subsequently matched and included in the final analysis (*n* = 40).

CCBE1 was selected for targeted evaluation because it had emerged as a gene of interest from prior work in our group, including an unpublished dataset in which CCBE1 expression was detected in PBMCs, and earlier transcriptomic analyses of endothelial colony‐forming cells (ECFCs) where CCBE1 was implicated in cardiovascular disease–related pathways [22]. Accordingly, the scRNA‐seq analysis in this study was designed as an exploratory assessment to determine whether CCBE1 expression could be replicated in PBMC subsets.

### 2.6 Statistical Analysis

Data are expressed as mean ± SEM for the specified number of experiments. Normality and variance tests were conducted prior to selecting the appropriate *t*‐test. For comparisons between two groups, parametric data were analyzed using Student's *T*‐test, whereas nonparametric data were analyzed using the Mann–Whitney *U* Test. Categorical variables are presented as frequencies and percentages, while continuous variables are reported as mean ± standard deviation for normally distributed data and median with interquartile range for nonnormally distributed data. Pearson's chi‐squared test was used to compare categorical variables, while Student's *t*‐tests were applied to continuous variables. Univariate linear regression was performed to assess for association between CAD severity and MitoSOX signature. Multivariable linear regression was performed, adjusting for sex age and SMuRFs (hypertension, diabetes mellitus, hypercholesterolaemia, smoking history). Statistical significance was set at *p* ≤ 0.05. All analyses were performed using GraphPad Prism (version 10.0.0, San Diego, CA, USA), R Studio (Version 4.4.2, Vienna, Austria) and Jamovi (version 2.3, Sydney, NSW, Australia).

## 3 Results

### 3.1 Baseline Characteristics of the Population

Among the 40 patients in the exploratory cohort, 25 subjects had CAD, and 15 subjects were No CAD controls. Ages between CAD (mean age: 61.8) and non‐CAD (mean age: 56.2) were similar. There were significantly more males with CAD compared to No CAD (*p* = 0.008). All patient baseline characteristics are shown in Table 1. All patients were nondiabetic, not current smokers, and had no history of percutaneous coronary intervention history or coronary artery bypass grafting.

**TABLE 1:** Clinical parameters according to CAD status of patients.

Characteristic	Whole cohort (*n* = 40)	Non‐CAD (CACS = 0) (*n* = 15)	CAD (CACS > 0) (*n* = 25)	*p*
Age, mean (SD)	59.7 (8.9)	56.2 (8.6)	61.8 (8.5)	0.054
Male, *n* (%)	16 (40.0)	2 (13.3)	14 (56.0)	0.008
Hypertension, *n* (%)	10 (26.3)	2 (13.3)	8 (34.8)	0.140
Hypercholesterolaemia, *n* (%)	17 (44.7)	6 (40.0)	11 (47.8)	0.640
Significant smoking history, *n* (%)	5 (11.4)	1 (5.6)	4 (15.4)	0.310
BMI—mean (SD)	27.2 (4.9)	25.9 (4.1)	28.1 (5.3)	0.180
0 SMuRFs BMI—mean (SD)	25.4 (4.0)	24.8 (4.4)	26.0 (3.8)	0.640
BMI ≥ 30 kg/m^2^, *n* (%)	10 (26.3)	4 (26.7)	6 (26.1)	0.970
Significant family history CAD, *n* (%)	17 (39.5)	5 (27.8)	12 (48.0)	0.180
SMuRFs—median (IQR)	1.0 (1.75)	1.0 (1.0)	1.0 (2.0)	0.060
0 SMuRFs, *n* (%)	12 (27.3)	5 (27.8)	7 (26.9)	0.950
Coronary artery calcium score—median, IQR	36.6 (339.9)	0 (0.0)	266.8 (538.8)	< 0.010
Calcified plaque present (CACS > 0), *n* (%)	25 (62.5)	0 (0.0)	25 (100.0)	< 0.010
Anti‐coagulant—*n* (%)	3 (7.9)	2 (13.3)	1 (4.3)	0.320
Anti‐platelet agent—*n* (%)	6 (15.8)	2 (13.3)	4 (17.4)	0.740
Statin—*n* (%)	19 (50.0)	7 (46.7)	12 (52.2)	0.740
β‐blocker—*n* (%)	3 (7.9)	1 (6.7)	2 (8.7)	0.820
ACE/ARB agent—*n* (%)	10 (26.3)	2 (13.3)	8 (34.8)	0.140

Abbreviations: ACE, angiotensin‐converting enzyme; ARB, angiotensin II receptor blockers; BMI, body mass index; CACS, coronary artery calcium score; CAD, coronary artery disease; SMuRFs, standard modifiable risk factors.

### 3.2 mROS Generation in Male Lymphocytes Have a Moderate Association to CACS Score

**FIGURE 2:**
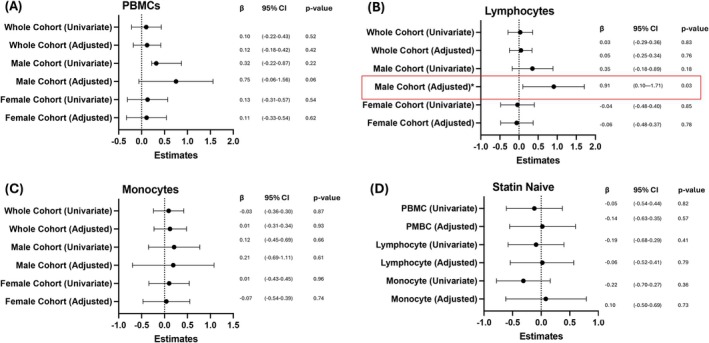
mROS production levels of PBMCs have an association with CAD severity. Univariate and adjusted (age, sex, smoking status, hypercholesteremia) standardized estimates of MitoSOX‐positive signature and CACS for each cohort in (A) PBMCs, (B) Lymphocytes, (C) Monocytes and (D) Statin naïve patients. *N* = 25 with CAD, *N* = 15 with No CAD. *N* = 8 with No CAD and Statin naïve, *N* = 13 with CAD and Statin naive.*p < 0.05.

Our primary objective was to study whether a mROS production phenotype exists in patients with or without CAD by performing flow cytometry analysis on PBMCs. In the exploratory cohort of 40 freshly isolated PBMCs, in 25 patients with CAD and 15 no CAD controls, no association was found (*R* ^2^ = 0.01, *p* = 0.52; Figure 2A) between MitoSOX Red signal and Log[CACS+1]. Next, we investigated sex‐specific differences and observed an association in the PBMCs of male patients with or without CAD. Here, we observed no association between Log[CACS+1] and MitoSOX Red signal (*R* ^2^ = 0.10, *p* = 0.22; Figure 2A). No association was observed within the female CAD or No CAD subcohort (Figure 2A). Subsequently, we performed multivariable linear regression and adjusted for priori covariates (age, sex, hypertension, hypercholesteremia, diabetes, smoking). When adjusted, there was no association between Log[CACS+1] and MitoSOX Red levels (adjusted *R* ^2^ = 0.29, *p* = 0.42; Figure 2A). For male patients with or without CAD, there was no association (adjusted *R* ^2^ = 0.08, *p* = 0.06; Figure 2A). No changes were observed for female patients with or without CAD (Figure 2A).

When investigating the lymphocyte subpopulation, there was no association (*R* ^2^ = 0.001, *p* = 0.83; Figure 2B) between Log[CACS+1] and MitoSOX Red levels. Within the male subcohort with varying CAD severity, there was no association between lymphocyte MitoSOX Red signal and Log[CACS+1] (*R* ^2^ = 0.13, *p* = 0.18; Figure 2B). No trend was observed for the female subcohort lymphocyte population of varying CAD severity (Figure 2B). When the association between Log[CACS+1] and lymphocyte MitoSOX Red levels was adjusted for priori covariates, there was no association (adjusted *R* ^2^ = 0.24, *p* = 0.76; Figure 2B). For the male subcohort of varying CAD severity, there was also a moderate, positive association between MitoSOX red signal and CACS (adjusted *R* ^2^ = 0.21, *p* = 0.0032; Figure 2B). No trends were observed for the female cohort based on CAD severity (Figure 2B).

Subsequently, we looked at the monocyte subpopulation; however, no significant association was detected in all groups (Figure 2C). Lastly, due to the pleiotropic effects of statins [23, 24], we investigated if there was any association within the statin naïve subcohorts and reported no differences (Figure 2C). In the lymphocyte subpopulation of the statin‐naïve group, no differences were also reported (Figure 2D). No differences were reported in the monocyte subpopulation either (Figure 2D). As we did not have enough male or female statin naïve patients, no analysis was conducted.

### 3.3 Higher Levels mROS Generation in Monocytes Are Slightly Associated With Lower Odds of Hypertension

**FIGURE 3:**
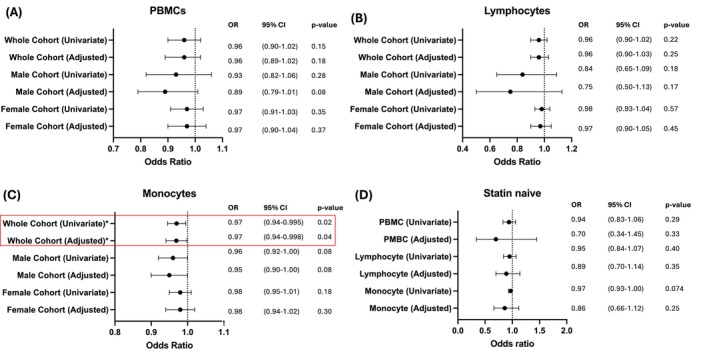
mROS production levels of monocytes have an association with hypertension. Univariate and multivariable adjusted (age, sex, smoking status, hypercholesteremia, coronary artery disease status) logistic regression of MitoSOX‐positive signature and hypertension for each cohort in (A) PBMCs, (B) Lymphocytes, (C) Monocytes and (D) Statin naïve patients. *N* = 10 with Hypertension, *N* = 28 with No Hypertension. *N* = 5 with Hypertension and Statin naïve, *N* = 14 with No Hypertension and Statin naïve. *p < 0.05.

Our secondary objective was to investigate mROS generation in PBMCs, lymphocytes, and monocytes and its association with SMuRFs (hypertension, hypercholesteremia, smoking history). We found no association in the whole cohort, male or female subcohorts for associations between mROS generation and PBMCs, lymphocytes (Figure 3A,B).

In monocytes, we found that there was a reduced odds ratio of hypertension associated with mROS generation (odds ratio: 0.97 [95% CI: 0.94–1.00; *p* = 0.02]; Figure 3C). When adjusted for covariates (age, sex, hypercholesteremia, diabetes, smoking history, and coronary artery disease), we found that in monocytes there was an odds ratio of 0.97 (95% CI: 0.94–1.00; *p* = 0.04; Figure 3C). Within the statin naïve cohort of patients with or without hypertension, no significant association was found in PBMCs, lymphocytes, or monocytes (Figure 3D).

### 3.4 mROS Generation Has no Association With Hypercholesteremia

**FIGURE 4:**
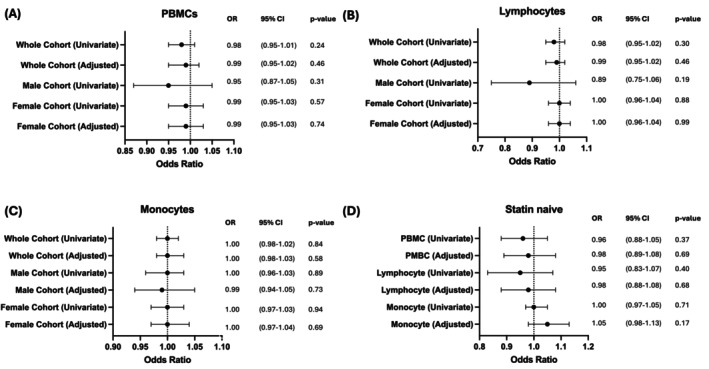
mROS production levels have no association with hypercholesteremia. Univariate and multivariable adjusted (age, sex, smoking status, hypertension, coronary artery disease status) logistic regression of MitoSOX‐positive signature and hypertension for each cohort in (A) PBMCs, (B) Lymphocytes, (C) Monocytes and (D) Statin naïve patients. *N* = 10 with Hypercholesteremia, *N* = 28 with No Hypercholesteremia. *N* = 5 with Hypercholesteremia and Statin naïve, *N* = 14 with No Hypercholesteremia and Statin naïve. *p < 0.05.

We next investigated if mROS generation in hypercholesteremia and report no significant association was associated in PBMCs, lymphocytes, or monocytes in all subgroups (Figure 4A–D). Analyses for the male cohort with or without hypertension in total PBMCs and monocyte populations were omitted due to low sample size and overfitting issues (Figure 4A,B).

### 3.5 Single‐Cell RNA Sequencing of Matched PBMCs

We recently reported a novel finding that expression of *CCBE1* (Collagen and calcium‐binding EGF domain‐containing protein 1)—a gene implicated in lymphangiogenesis and vascular remodeling—is significantly upregulated in endothelial colony‐forming cells (ECFCs) derived from patients with CAD [22]. Given this, we sought to determine whether mitochondrial reactive oxygen species (mROS) production is associated with *CCBE1* expression. Although CCBE1 has no known canonical role in PBMCs, it has been detected in bulk RNA sequencing data from 29 purified immune cell types [25]. Therefore, we investigated whether *CCBE1* expression in PBMCs could serve as a marker for cardiovascular risk.

**FIGURE 5:**
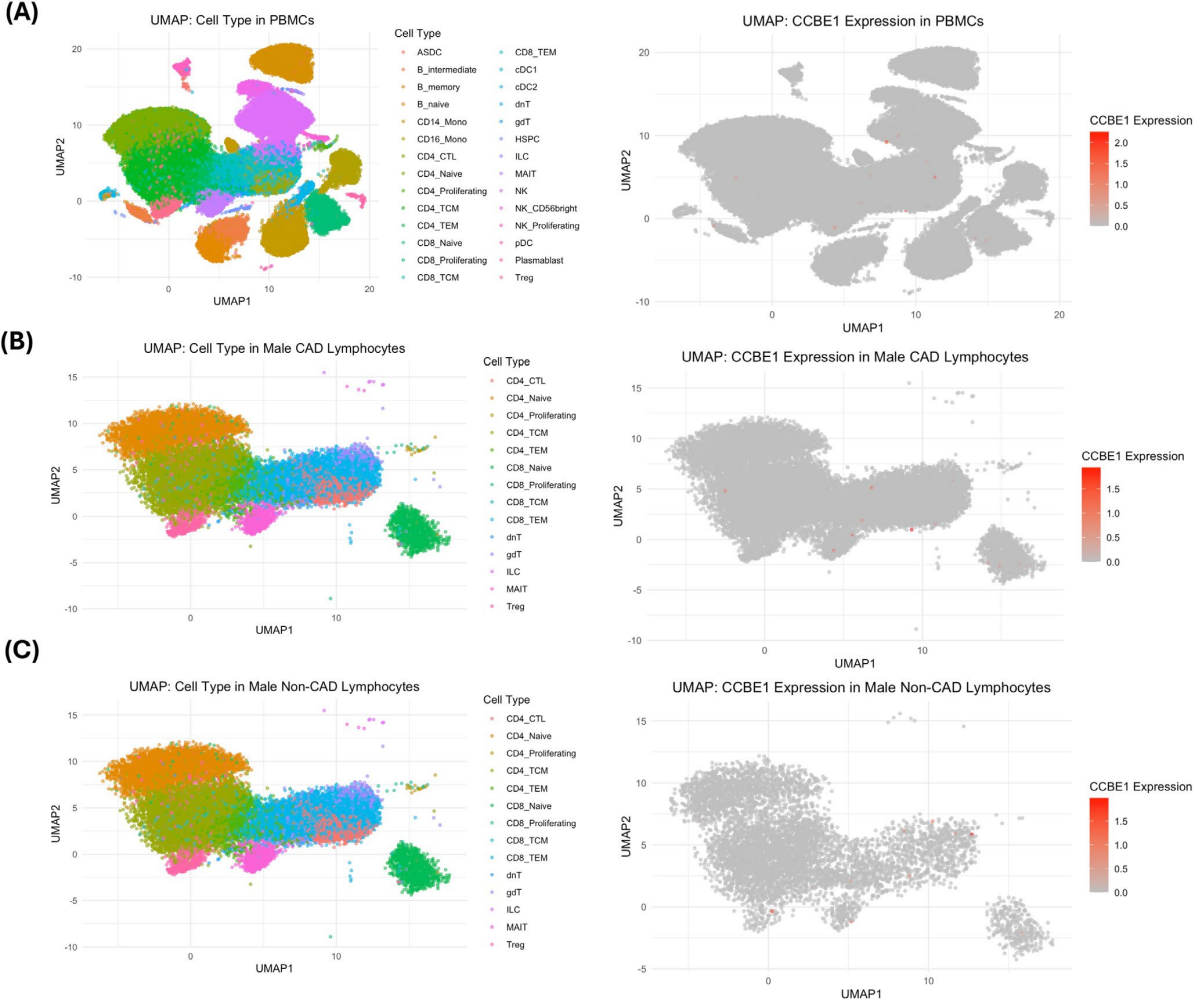
CCBE1 expression across immune cell types in patients with and without CAD using single cell RNA‐seq. (A) UMAP visualization of CCBE1 expression in matched patient peripheral blood mononuclear cells (PBMCs) from scRNA‐seq data. (B) CCBE1 expression in male CAD patient lymphocytes. (C) CCBE1 expression in male non‐CAD patient lymphocytes. Statistical comparisons were performed with Fisher's exact test.

Using single‐cell RNA sequencing (scRNA‐seq) data from matched patient samples, we visualized *CCBE1* expression across immune cell types. Overall, CCBE1 expression was low (Figure 5A), with the highest expression observed in natural killer (NK) cells (0.57%). We focused our CCBE1 scRNA‐seq analysis on male lymphocytes because our primary results indicated that mROS generation in this cell population exhibited a moderate association with CACS. This motivated a targeted investigation of whether CCBE1 expression within male lymphocyte subpopulations could provide mechanistic insight into this relationship. Among CAD patients, CD4^+^ cytotoxic T lymphocytes (CD4^+^ CTLs) exhibited the highest CCBE1 expression (0.51%), whereas gamma delta T cells (γδ T cells) showed the highest expression (0.94%) in non‐CAD individuals (Figure 5B,C; Data S1). However, no statistically significant differences were found in CCBE1‐expressing lymphocyte subpopulation proportions between CAD and non‐CAD groups (Data S1).

To further assess the relationship between *CCBE1* expression and mROS generation, we analyzed hypertensive and nonhypertensive matched patients. In this comparison, CCBE1 expression was absent in both CD14^+^ and CD16^+^ monocytes (Figure S1), suggesting no association between CCBE1 and mROS levels in monocytes.

## 4 Discussion

Unmet needs in the early detection and diagnosis of CAD beyond traditional risk factors remain at the forefront of disease prevention and risk. With oxidative stress known to play a key role in the development and progression of atherosclerosis [7] in addition to lymphocytes and monocytes [26, 27], the use of circulating PBMCs in combination with mROS generation suggests that it may be a promising and relevant tool in identifying the disease early. As CAD is widely considered a chronic, long‐term inflammatory condition, we initially anticipated that such persistent inflammatory activity might be detectable in PBMC‐mitochondrial redox phenotypes. For this reason, we envisioned that the BioHEART‐CT cohort—representing individuals with stable symptoms undergoing CT‐based assessment—would be an appropriate population in which to interrogate PBMC mROS in relation to disease.

However, in this exploratory cohort of chronic, stable CAD patients, we did not find consistent or robust associations between PBMC mROS and the presence or severity of CAD, nor with most traditional modifiable risk factors. This suggests that long‐term inflammatory burden alone may not be sufficient to generate detectable mitochondrial redox changes in circulating immune cells. Instead, mitochondrial ROS responses may be more pronounced during unstable or high‐inflammatory states, such as acute coronary syndromes, where immune activation and oxidative signaling are dynamically amplified. As such, the stable nature of the BioHEART‐CT cohort may limit the observable effect, and PBMC mROS may not serve as a sensitive biomarker for established, stable disease.

Although small subgroup‐specific associations were observed—such as a moderate relationship between lymphocyte mROS and CACS in males, and a modest inverse association between monocyte mROS and hypertension—these signals were borderline, inconsistent across analyses, and should be considered hypothesis‐generating rather than clinically meaningful. As such, our data suggest that PBMC mROS is unlikely to function as a discriminatory biomarker for existing CAD in stable populations.

This interpretation aligns with previous literature showing that PBMC mROS alterations are more evident in acute or unstable cardiovascular conditions. mROS production in the PBMCs of patients with acute coronary syndrome compared to healthy controls was increased [28]. The absence of meaningful associations in our BioHEART‐CT cohort highlights that PBMC mitochondrial redox activity may reflect dynamic inflammatory states rather than chronic quiescent disease.

Interestingly, our study is the first to report the association between mROS generation and freshly isolated lymphocytes from CAD severity. In circulating monocytes, which are precursors to macrophages, there did not have a significant association. This is interesting as canonically it is believed that macrophages produce mROS in a pro‐atherosclerotic environment [29, 30, 31]. Previously, the mitochondrial dynamics and bioenergetics of monocytes had not been fully elucidated [32].

Moreover, we report a sex‐linked association with male patients exhibiting a significant association. One potential reason is that the rate of mROS production is significantly lower in females compared to males [33]. A study showed that the mitochondria of female rats exhibited higher levels of reduced glutathione, Mn‐superoxide dismutase expression, and glutathione peroxidase, which ultimately confers protection against oxidative stress [33].

The mechanisms by which mROS production is increased in lymphocytes during atherosclerosis are not fully understood. One hypothesis is that ROS originating from the myocardium might stimulate mROS production in lymphocytes via the self‐fulfilling cycle of ROS‐induced ROS generation [34]. Another explanation is the presence of inflammatory factors such as IL‐1β and IFN‐⍺ [35]. For instance, chronic heart failure (CHF) patients demonstrated associations between mROS production and heart failure severity (plasma BNP levels) [36].

In the literature, mROS generation is typically reported to increase and contribute to hypertension pathogenesis through oxidative damage [37]. In contrast, our data show that increased mROS generation is slightly associated with lower odds of hypertension in monocytes, although the effect size is small. As this directionality is not supported by existing evidence, we interpret the finding as novel and hypothesis‐generating. Given the exploratory nature of our study—using ex vivo assays in a relatively small cohort—the stability and direction of this association should be interpreted with caution until validated in larger, more statistically powered cohorts.

Importantly, although the association reached statistical significance, the magnitude of the reduction was modest. This raises the question of clinical significance. Small effect sizes—particularly in mechanistic biomarkers such as mROS—may not translate into useful clinical discrimination at the individual‐patient level. It is therefore unlikely, based on the present data alone, that monocyte mROS would serve as a robust standalone biomarker for hypertension risk. Instead, its value may lie in contributing to multiparameter signatures that reflect broader mitochondrial or immuno‐metabolic phenotypes relevant to cardiovascular risk. Additionally, given that oxidative signaling can have context‐dependent, compensatory, or cell‐type–specific roles, the biological relevance of a small mROS shift in monocytes should be interpreted cautiously.

Overall, these findings should be viewed as hypothesis‐generating. Larger cohorts, longitudinal designs, and integration with vascular function or circulating oxidative markers will be essential to determine whether monocyte mROS has any meaningful clinical utility in hypertension assessment, or whether the observed association represents biological noise, compensatory signaling, or unmeasured confounding.

Taken together, these findings emphasize that cross‐sectional PBMC mROS measurements may not provide reliable diagnostic information in stable CAD. Nonetheless, mROS could still hold relevance in different clinical contexts. mROS‐related responses may be more pronounced during episodes of plaque instability, systemic inflammation, or acute coronary syndromes, where oxidative signaling is dynamically altered. Therefore, future longitudinal studies assessing whether PBMC mROS predicts recurrent or future cardiovascular events—particularly in unstable or high‐risk populations—are warranted and may better capture the prognostic potential of mitochondrial redox phenotypes.

Several strengths of this study should be highlighted. The use of PBMCs from the BioHEART‐CT study is vastly superior compared to the use of ECFCs as previously utilized [6, 10, 11]. Unlike ECFCs, PBMCs are easily isolated and can be screened almost immediately for the detection of CAD and other cardiovascular diseases. This, combined with the strengths of the BioHEART‐CT study, allows for the deep phenotyping of patient cell lines for the use of biomarker and drug discovery. Nevertheless, some limitations of this study should be acknowledged. The most significant limitation is the specificity of MitoSOX, which previously has been shown to be nonspecific [38]. Future studies are needed to incorporate more specific mitochondrial probes like the newly developed MitoNeoD [39]. Another key limitation of this study is the relatively small sample size, which limits statistical power and likely contributes to the wide confidence intervals observed. As this was an exploratory study without a prior power calculation, the findings should be interpreted cautiously and require validation in larger, prospectively powered cohorts to determine whether subtle but meaningful associations between PBMC mROS and CAD truly exist.

## Author Contributions

W.E.L. and G.A.F. wrote the manuscript; W.E.L. designed the research; W.E.L., A.H., E.R.S., E.S.‐Z., B.B., T.V.N., S.M.G., J.E.P., M.P.G., and G.A.F. performed the research. A.H., E.S.‐Z., B.B., and J.E.P. contributed new reagents/analytical tools.

## Funding

G.A.F. reports grants from the National Health and Medical Research Council Australia (GNT2018194, GNT2005791) and the NSW Office for Health and Medical Research (OHMR) (H21/174585). The BioHEART study has received support from a combination of grants including from the Ramsay Teaching and Research Foundation, BioPlatforms Australia, the Vonwiller Foundation, and Heart Research Australia.

## Conflicts of Interest

G.A.F. has received grant support from the National Health & Medical Research Council (Australia), Abbott Diagnostic, Sanofi, Janssen Pharmaceuticals, and NSW Health. G.A.F. has received personal fees from CSL and CPC Clinical Research. G.A.F. serves as a Board Member for the Heart Foundation, President of the Australian Cardiovascular Alliance, Founding Director/CMO of Prokardia, and CSO of CAD Frontiers. G.A.F. has a patent “Patent Biomarkers and Oxidative Stress” which was awarded in the USA in May 2017 (US9638699B2) and licensed to Northern Sydney Local Health District. All other authors declared no competing interests for this work.

## Supporting information


**Data S1:** cts70469‐sup‐0001‐supinfo.docx.
