# Supplementary material for: Increased mROS Generation Associates With Cardiovascular Risk in BioHEART‐CT PBMCs
Source: Clin Transl Sci. 2026 Jan 18;19(1):e70469. doi: 10.1111/cts.70469 (PMC12813273; doi:10.1111/cts.70469)
Supplement: Supplementary file 1 — Data S1: cts70469‐sup‐0001‐supinfo.docx. [file CTS-19-e70469-s001.docx]

Figure S1. *Assessment of CCBE1 expression in monocyte subsets in relation to hypertension status.* CCBE1 expression was evaluated in CD14⁺ (classical) and CD16⁺ (non-classical) monocyte populations from hypertensive and non-hypertensive matched patients using scRNA-seq data. No detectable CCBE1 expression was observed in either monocyte subset, suggesting a lack of association between CCBE1 expression and mitochondrial ROS generation in these cell types.
